# Carrageenan nasal spray in virus confirmed common cold: individual patient data analysis of two randomized controlled trials

**DOI:** 10.1186/2049-6958-9-57

**Published:** 2014-11-12

**Authors:** Martin Koenighofer, Thomas Lion, Angelika Bodenteich, Eva Prieschl-Grassauer, Andreas Grassauer, Hermann Unger, Christian A Mueller, Tamás Fazekas

**Affiliations:** St. Anna Children’s Hospital, Kinderspitalgasse 6, 1090 Vienna, Austria; Department of Otorhinolaryngology, Medical University of Vienna, Waehringer Guertel 18-20, 1090 Vienna, Austria; Children’s Cancer Research Institute and Lab DiaLabordiagnostik, Zimmermannplatz 8, 1090 Vienna, Austria; Marinomed Biotechnologie GmbH, Veterinaerplatz 1, 1210 Vienna, Austria; Laboratory of Tropical Veterinary Medicine, Veterinary University Vienna, Veterinaerplatz 1, 1210 Vienna, Austria

**Keywords:** Carrageenan, Common cold, Coronavirus, Respiratory disease, Rhinovirus, Influenza, Virus

## Abstract

**Background:**

Clinical trials applying iota-carrageenan nasal spray have previously shown to reduce duration of virus-confirmed common cold. The present study pooled data of two similar clinical trials to provide further evidence for the antiviral effectiveness of carrageenan.

**Methods:**

Individual patient data were analyzed from two randomized double blind placebo controlled trials assessing the therapeutic effectiveness of carrageenan nasal spray in acute common cold. Patients with virus-confirmed common cold (n = 254, verum 126, placebo 128) were included and the following parameters were appraised: duration of disease, number of patients with relapses, number of respiratory viruses and viral titers at inclusion (visit 1) compared to days 3–5 (visit 2).

**Results:**

Carrageenan treated patients showed a significant reduction in duration of disease of almost 2 days (p < 0.05) as well as significantly fewer relapses during 21 days of observation period (p < 0.05). The virus clearance between visit 1 and visit 2 was significantly more pronounced in the carrageenan group (p < 0.05). In both studies, virus-confirmed common cold was caused by three main virus subtypes: human rhinovirus (46%), human coronavirus (25%) and influenza A (14%) virus. Carrageenan nasal spray showed significant antiviral efficacy in all three virus subgroups, the highest effectiveness was observed in human corona virus-infected patients. The reduced duration of disease was 3 days (p < 0.01) and the number of relapses was three times less (p < 0.01) in carrageenan treated corona-virus-infected patients compared to control patients.

**Conclusions:**

Administration of carrageenan nasal spray in children as well as in adults suffering from virus-confirmed common cold reduced duration of disease, increased viral clearance and reduced relapses of symptoms. Carrageenan nasal spray appeared as an effective treatment of common cold in children and adults.

**Trial registration:**

Pooled data from ISRCTN52519535 and ISRCTN80148028

## Background

Acute viral upper respiratory tract infection, also known as common cold, is the most frequently observed infectious disease in human beings. Children get four to eight upper respiratory infections per year and adults suffer from two to four episodes per year [1]. In the majority of cases common cold is caused by respiratory viruses such as rhinovirus, coronavirus, parainfluenza, influenza, respiratory syncytial virus, adenovirus, enterovirus and metapneumovirus [2–4]. Although common cold is a self-limiting disease, the symptoms such as runny nose, nasal congestion, sneezing, cough, sore throat, malaise and fever are troublesome, leading to more than 20 million doctor visits and 40 million lost school and work days per year [5]. Despite the enormous economic and social burden of common cold, an effective treatment is still not available.

As common cold is caused by diverse viruses, an effective therapeutic substance should exhibit a broad antiviral capacity and should not lead to resistance formation. Carrageenans may represent such an option. Carrageenans belong to a family of linear, sulfated polysaccharides which are found in some species of red seaweed. Food, cosmetic, and pharmaceutical industry use carrageenans extensively as emulsifying and binding agent for products like ice cream, various gels, toothpaste and others [6]. But carrageenans also revealed antiviral activity against a range of animal viruses [7] and are even used to prevent sexual transmitted viral infections as a component of spermicides [8]. Furthermore, *in vitro* and *in vivo* studies have recently shown that carrageenans are potent inhibitors of papilloma virus [9], human rhinovirus [10], influenza A virus [11], respiratory syncytial virus and also of human enterovirus 71 [12].

Three randomized controlled clinical trials (RCT) showed the superior symptomatic benefit [13] and antiviral efficacy of carrageenan containing nasal spray [14, 15] in patients with common cold. The current study aims to provide a deeper insight into the results of the two larger RCTs. Carrageenan is a large polymer which does not permeate the nasal mucosa and the antiviral effect is based on a physical mode of action only. Therefore, this host-independent mode of action allows pooling the data from a study in children and adults which would not be acceptable for a substance exhibiting a pharmacological action. Subgroups of individual viruses in the two trials were quite small, so pooling of data allowed the analysis of subgroups of the most frequently occurring viruses. In addition, it was investigated if the broad antiviral activity observed *in vitro* against enveloped viruses (influenza and corona virus) and non-enveloped viruses (rhinovirus) is also reflected in the clinical data. Therefore, data of two similar trials were merged and an additional analysis regarding the antiviral effectiveness of carrageenan nasal spray in patients with virus-confirmed common cold was performed.

## Methods

Individual patient data from two phase III randomized, double-blind, placebo-controlled trials were pooled for this analysis [14, 15]. Main characteristics of these trials are given in Table 1.

**Table 1:** **Characteristics of randomized controlled studies included into efficacy analysis of carrageenan nasal spray in common cold**

Trial	Number of patients	Population	Main inclusion criteria	Duration of nasal application of carrageenan or placebo	Outcomes	Follow-up
Fazekas T. et al. 2012 [14]	213	1-18 years	Duration of symptoms since 36 h	7 days 3 times per day	Symptoms severity; number of days without symptoms; viral load	21d
Ludwig M. et al. 2013 [15]	211	≥18 years	Duration of symptoms since 48 h	7 days 3 times per day	Duration of disease; symptoms severity; viral load	21d

Both trials were approved by Independent Ethics Committees of the participating centres and conducted according to the Declaration of Helsinki and applicable local regulations.

Main inclusion criteria in both trials were the presence of early symptoms of common cold, with symptoms up to 36 hours before enrollment in the children’s trial and up to 48 hours in the adult trial. The severity of the cold was measured using a symptom score [16]. The score was calculated by summing 8 symptoms (headache, muscle ache, chilliness, sore throat, nasal obstruction, nasal discharge, cough, and sneezing) with each item rated 0 = absent, 1 = mild, 2 = moderate and 3 = severe. Patients with a symptom score of 1–9 (children’s trial) or 2–9 (adult’s trial) at the time of inclusion were enrolled in the study. Investigators and patients were masked to treatment and the test spray and matching placebo were indistinguishable. Patients were randomly assigned using a permuted block schedule (size four). After randomization, patients received treatment with either iota-carrageenan nasal spray (0.12% iota-carrageenan/0.5% sodium saline; “carrageenan”) or placebo (0.9% sodium saline; “placebo”) three times per day, for 7 days. Presence of common cold symptoms were recorded in daily diaries till day 21 after inclusion in trials.

Nasal lavage samples were collected on day 1 (visit 1, V1) before the first application of the medication and either on days 3 to 5 [14] or days 3 to 4 [15] respectively (visit 2, V2) during treatment. In the second trial [15], nasal lavage samples were also obtained on study days 10 or 11, after treatment. If one of the third samples was virus positive, patients were also included into analysis.

Molecular screening and quantitative assessment of viral load in nasal lavage samples were performed for influenza virus types A (InfA) and B (Inf B), respiratory syncytial virus (RSV), parainfluenza viruses (PIV) types 1–3, human rhinovirus (hRV), human metapneumovirus (MPV), coronavirus types OC43 and 229E (hCV). A patient was considered to be virus-positive if any virus was detected at least in one sample. For detailed information on the methods used see previous publications [14, 15].

According to study objectives, patient data from all virus-positive patients were included into the pooled analysis. For the assessment of clinical effectiveness the following parameters were determined: (1) duration of disease and (2) number and frequency of relapses. The duration of disease was defined as the time until the last day with symptoms of common cold. Relapses were defined as re-occurrence of symptoms when the patient reported having common cold symptoms again after having reported at least one day without symptoms.

For assessment of antiviral effectiveness the following variables were selected: (1) number of different viruses detected at V1 and V2, (2) change of viral titers from V1 to V2 and (3) number of patients with viruses at V1 and V2. Similar to adverse events which can occur several times in one patient, several common cold viruses can infect one patient at the same time. Consequently, for the calculation of the difference of viral titers between V1 and V2 the concept of virus events was applied, this means that calculations are performed for each virus individually. Gender and age were assessed as important demographic co-variables. The analysis of these parameters was performed for the virus-positive (VP) population and for patient subgroups that were positive for a specific virus of the most frequently detected ones (i.e., hRV, hCV and InfA).

All findings after the screening visit were documented as adverse effects (AEs) except for any symptoms attributed to common cold (sneezing, runny nose, nasal obstruction, sore throat, cough, headache, fatigue and chilliness). Nevertheless, if such symptoms were of marked intensity or deemed to be not related to the study disease, these were recorded as AEs.

Statistical analysis was performed using statistical IBM software package SPSS Version 20.0. Analysis of duration of disease was done by using the log rank test. All data sets were checked for normal distribution by the Kolmogorov-Smirnov-Test prior to analysis. Binary or categorical data (e.g. presence or absence of viruses, number of patients with adverse events) were analysed by the Chi-Square test. Viral titer in nasal lavage samples and changes in viral titers between visits were analysed using non-parametric Mann–Whitney U-test due to the fact that the distribution of data was not normal and in addition the data set was highly skewed. In the dataset for this analysis, all viruses detected were included and each virus was accounted for individually.

## Results

### Patients

**Figure 1:**
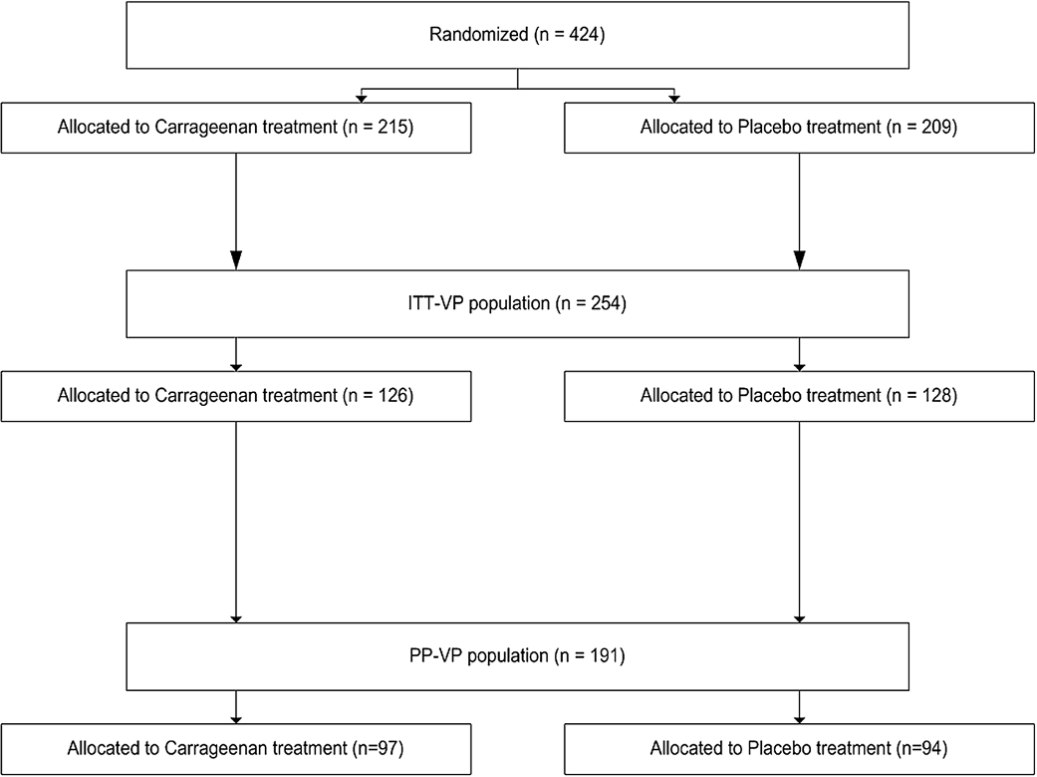
**Distribution of patients included in the pooled analysis.**

In the two double-blinded randomized controlled trails 254 virus-positive patients were enrolled in the intention-to-treat (ITT) population. Out of them, 126 patients were randomized into carrageenan treatment, whereas 128 patients were allocated to placebo treatment. In the per-protocol (PP) population remained 191 patients divided in 97 patients in carrageenan group and 94 patients in placebo group (Figure 1).

Demographic data are shown in Table 2. The virus positive ITT population consisted of 136 females (59 carrageenan and 77 placebo) and 118 males (67 carrageenan and 51 placebo) (Table 2). A statistical significant difference was recognized in distribution of males and females in carrageenan and placebo groups in ITT population. This difference derived mainly from the study undertaken in children (mean age 5 years) [14]. Nevertheless, a statistical adjustment analysis was performed but didn’t show any difference in results (data not shown). No distribution differences were seen in any other patient population, e.g. PP population, or in one of the three virus infected subgroups (data not shown).

**Table 2:** **Demographic characteristics of patients**

	ITT population					PP population				
	Carrageenan		Placebo			Carrageenan		Placebo		
	n = 126	%	n = 128	%	p	n = 97	%	n = 94	%	p
Male	67	53	51	40		54	56	41	44	
Female	59	47	77	60	0.033*	43	44	53	56	0.096
	Mean	SD	Mean	SD		Mean	SD	Mean	SD	
Age, years	17	16	18	17	0.9**	19	16	20	17	0.15

*Chi-square test.

**t-test.

SD, Standard deviation.

### Duration of symptoms and clinical effectiveness

**Figure 2:**
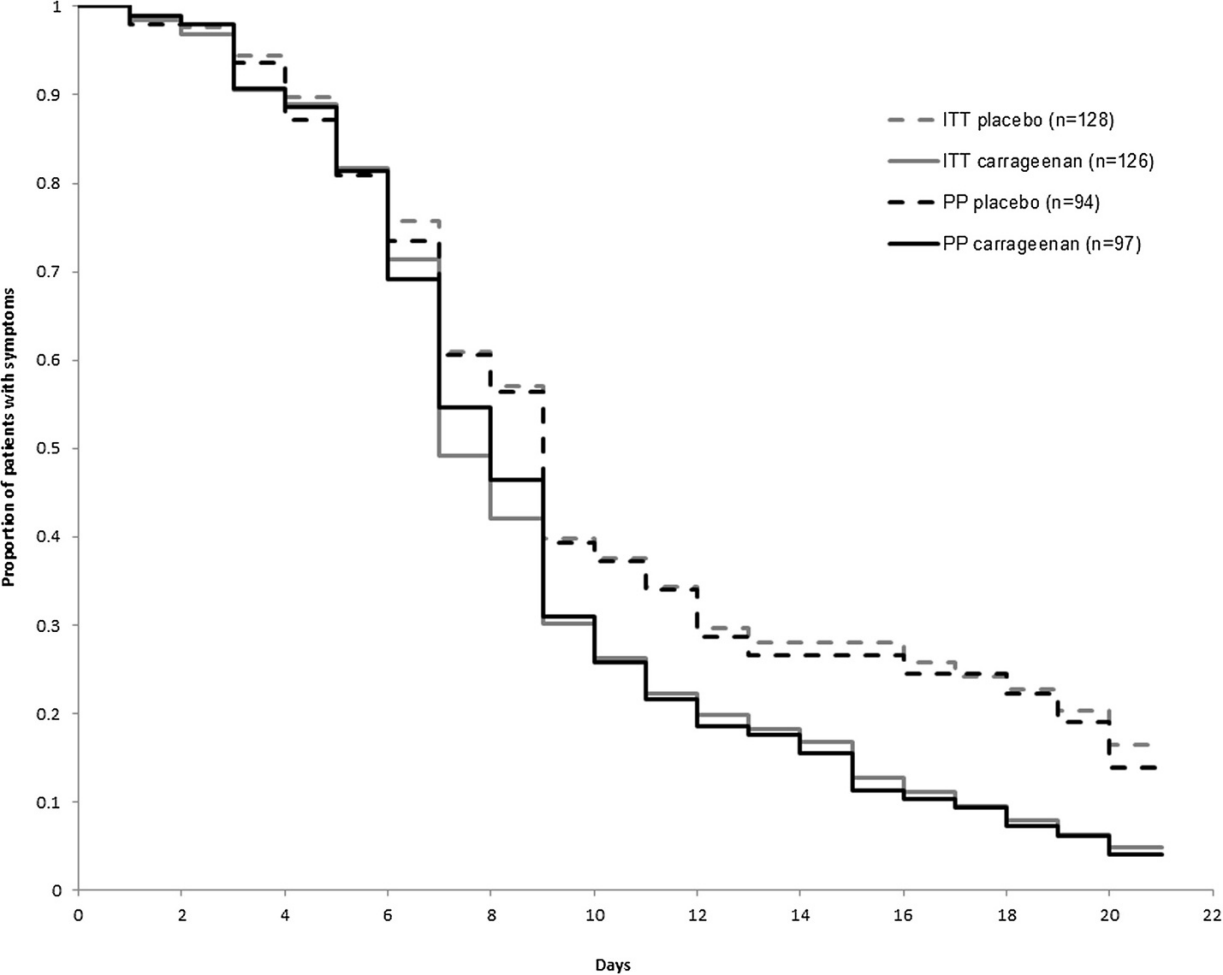
**Duration of common cold symptoms in ITT and PP groups.** Duration of common cold symptoms in virus-positive patients treated with carrageenan or placebo in ITT or PP population, respectively (ITT: carrageenan vs. placebo p = 0.002; PP: carrageenan vs. placebo p = 0.016).

At inclusion, intensity of common cold symptoms (symptom score) was similar for carrageenan (6.42 ± 0.18) and placebo (6.59 ± 0.16) treated patients, in ITT population (p = 0.485). Similarly, there was no difference in PP population in the respective groups, carrageenan (6.47 ± 0.2) and placebo (6.43 ± 0.18). Duration of common cold symptoms was significantly reduced in carrageenan treated patients compared to placebo treated ones, in both the ITT and the PP population. The average duration of disease was diminished by 1.9 days in ITT carrageenan group and by 1.7 days in PP carrageenan group compared to the respective placebo groups (ITT: p = 0.002; PP: p < 0.016) (Figure 2).

### Relapses

**Figure 3:**
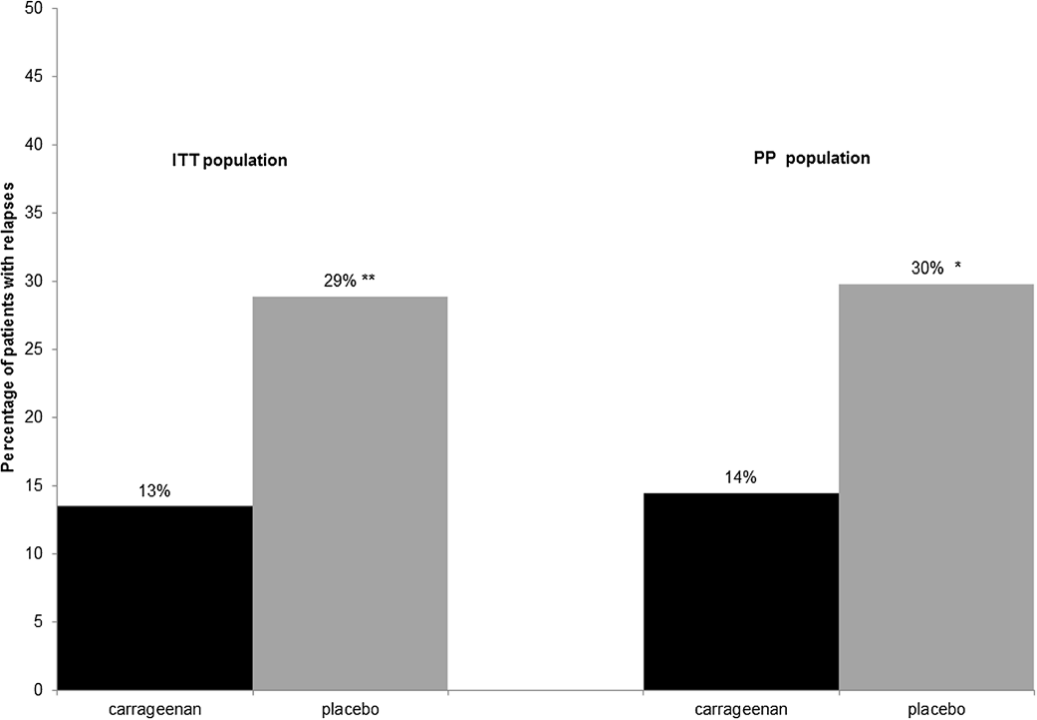
**Percentage of patients with relapses during the 21 days observation period.** Relapses in carrageenan and placebo treated groups presented in ITT and PP population. (* p < 0.05; ** p < 0.01).

During the 21 days of observation, relapses were observed significantly more frequently in placebo groups than in carrageenan treated patients, irrespectively of ITT or PP population (ITT: p = 0.003; PP: p = 0.01) (Figure 3). As some patients had more than one relapse during observation period, the average number of relapses per patient was calculated. The average number of relapses per patient was 0.17 in carrageenan treated patients compared to 0.45 in placebo treated patients in ITT population (p = 0.002). Likewise, the average number of relapses per patient was significantly higher in placebo group, namely 0.38 compared to 0.15 in carrageenan group in PP population during observation period (p = 0.01).

### Analysis of antiviral efficacy

For the analysis of antiviral effectiveness, patients who were virus-positive on visit 1 (day 1), visit 2 (days 3–5) or on both visits were included. Patients who missed visit 2 were excluded from the analysis because of incomplete data. Each detected virus was taken as a separate event. At inclusion, irrespectively of ITT or PP population, average viral titers (measured as log[x + 1]) of obtained real time PCR copy numbers were similar in carrageenan (ITT: 4.82 ± 0.2; PP: 4.42 ± 0.2) and placebo (ITT: 4.42 ± 0.2; PP: 4.66 ± 0.2) groups. A significant decrease in viral titers was observed at visit two in all groups. However, in ITT population the decrease in viral titers was significantly more prominent in carrageenan group (−2.2 log[x + 1]) than in placebo group (−1.1 log[x + 1]; p = 0.022). The data indicated that treatment with iota-carrageenan resulted in a reduction of viral titer at visit 2 which was an order of magnitude of more than 90% stronger when compared with placebo treatment. Similarly, the difference in virus titers between visit 1 and visit 2 was significantly higher in carrageenan group compared to placebo group in PP population (carrageenan: −2.25, placebo: −1.2; p = 0.044) (Table 3).

**Table 3:** **Viral titers at V1 and V2 (log[x + 1]) in respective groups**

Population	Treatment	Number of virus events	Visit	Virus titer (median (25%; 75% percentile)	Significance (Wilcoxon Signed Rank Test)	Reduction in viral titers (difference V1-V2) (median (25%; 75% percentile)	Significant difference between carrageenan and placebo (Mann- Whitney U- test)
ITT	Verum (n = 116)	154	V1	4.64 (3.41;6.57)			p = 0.022
	Verum (n = 116)	154	V2	3.61 (0;4.97)	p < 0.001	−2.2 (−3.47;-0.26)	
	Placebo (n = 120)	163	V1	4.49 (3.24;6.3)			
	Placebo (n = 120)	163	V2	3.82 (0;4.85)	p < 0.001	−1.14 (−3.31;0.31)	
PP	Verum (n = 87)	115	V1	4.66 (3.4;6.54)			p = 0.044
	Verum (n = 87)	115	V2	3.63 (0;4.83)	p < 0.001	−2.25 (−3.47;-0.57)	
	Placebo (n = 87)	113	V1	4.99 (3.35;6.49)			
	Placebo (n = 87)	113	V2	3.98 (2.24;4.89)	p < 0.001	−1.2 (−3.24;0.21)	

**Figure 4:**
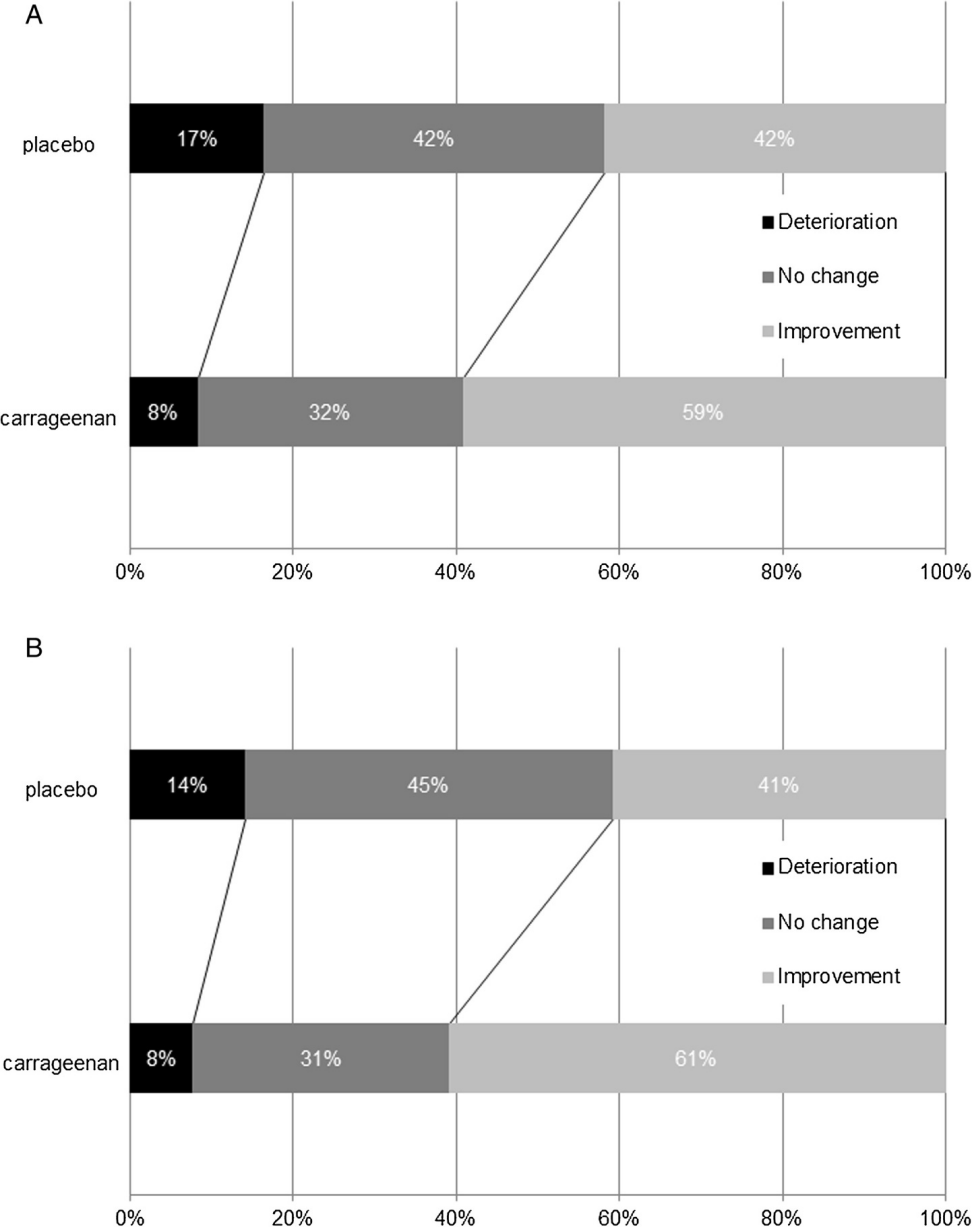
**Percentage of patients with dynamic changes in viral status between visit 1 and visit 2.** “Deterioration” was defined as > 50-fold increase of viral titer at visit 2 or detection of a new virus which was not revealed at visit 1. “Improvement” was defined as a > 50-fold decrease of viral titer at visit 2 or the elimination of a virus which was present at visit 1. “No change” was defined as a viral titer between 1:50 to 50:1 at visit 2 compared to visit 1. **A**: a ITT population p = 0.005 **B**: PP population p = 0.009.

Since in some patients more than one virus was detected, treatment groups were compared with regard to number of viruses per patient on visits 1 and 2. Whereas at baseline, groups did not differ significantly, at visit 2 numbers of viruses per patient were significantly lower in the carrageenan group. Accordingly, the change in the number of viruses per patient between visit 2 and visit 1 was more pronounced in the carrageenan group: on average, a placebo patient cleared 0.15 ± 0.07 viruses, while carrageenan treated patients lost 0.37 ± 0.07 viruses in ITT population (p = 0.02). Similarly, in PP population virus clearance was 0.14 ± 0.7 in placebo group versus 0.38 ± 0.7 in carrageenan group (p = 0.025). In carrageenan treated patients a significant increase of virus-free patients was observed between visit 1 and visit 2 in ITT as well as in PP population. In contrast, placebo treated patients showed no difference in virus-free individuals between visit 1 and visit 2 (Table 4). Figure 4 shows the percentage of patients with either a deterioration (defined as > 50-fold increase of viral titer at visit 2 or detection of a new virus which was not revealed at visit 1) an improvement (defined as a >50-fold decrease of viral titer at visit 2 or the elimination of a virus which was present at visit 1) or no change (defined as a viral titer between 1:50 to 50:1 at visit 2 compared to visit 1) in virus status between visit 1 and visit 2. Significantly more patients revealed an improvement in viral status between visit 1 and visit 2 in carrageenan treated patients compared to placebo treated ones, in ITT as well as in PP population. (ITT: p = 0.005; PP: p = 0.009, Figure 4).

**Table 4:** **Change in number of virus-free patients from visit 1 to visit 2 in ITT and PP population**

Patients	Group	Virus acquired	Virus lost	Significance
ITT (n = 236)	Placebo (n = 120)	11 (9%)	18 (15%)	n.s.
	Carrageenan (n = 116)	4 (3%)	27 (23%)	p < 0.001
PP (n = 174)	Placebo (n = 87)	5 (6%)	12 (14%)	n.s.
	Carrageenan (n = 87)	3 (3%)	20 (23%)	p < 0.001

McNemar test within-group comparison.

In the two pooled studies the following viruses were detected most frequently: hRV (142 patients), hCV (types OC43 or 229E, 78 patients) and InfA (43 patients). Patients positive for different viral types were distributed evenly between carrageenan and placebo treatment arms (Table 5).

**Table 5:** **Percentage of patients being positive for different types of viruses in ITT population**

Virus								
Group	hRV	hCV**	InfA	InfB	MPV	PIV***	RSV	Total
Verum	45 %	27 %	13 %	5 %	6 %	2 %	2 %	145
Placebo	47 %	24 %	14 %	7 %	6 %	2 %	1 %	161

**Patients positive for hCV OC43 or 229E or both.

***Patients positive for PIV types 1, 2 or 3.

**Figure 5:**
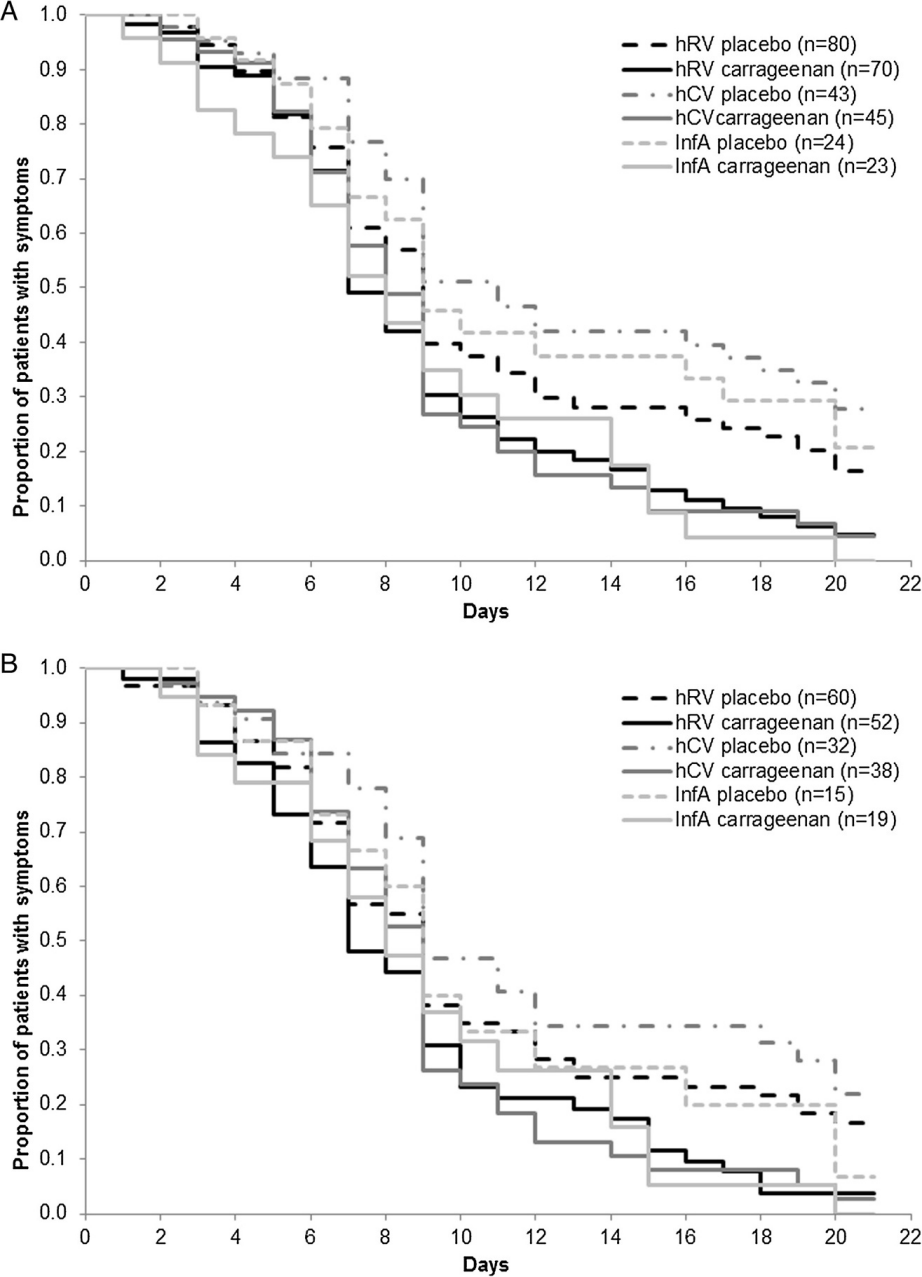
**Duration of common cold in carrageenan or placebo treated patients in hRV, hCV and InfA subpopulation. A**: Duration of common cold in ITT group (p = 0.019; p = 0.001; p = 0.02, respectively). **B**: Duration of common cold symptoms in carrageenan or placebo treated patients in hRV, hCV and InfA subpopulation in PP group (p = 0.041; p = 0.009; p = 0.27; respectively).

Subgroup analysis was done for the three most common virus types in the pooled population, hRV, hCV and InfA virus, respectively. In the ITT population, the duration of common cold disease was significantly reduced in all three virus subgroups treated with carrageenan. This effect was most pronounced in hCV infected patients treated with carrageenan where the reduction of duration was almost 4 days in ITT population (p < 0.01) and 3 days in PP population (p < 0.01) (Table 6 and Figure 5).

**Table 6:** **The average duration of common cold disease in patients of different virus subgroups in days**

Patients	Group	hRV	hCV	InfA
ITT	Carrageenan	8.8 ± 0.6	9.02 ± 0.7	8.7 ± 1.0
	Placebo	10.7 ± 0.7	12.95 ± 0.99	12.0 ± 1.2
	Difference	1.9 days*	3.9 days**	3.3 days*
PP	Carageenan	8.7 ± 0.7	9.1 ± 0.7	9 ± 1.1
	Placebo	10.5 ± 0.8	12.2 ± 1.1	10.7 ± 1.5
	Difference	1.8 days*	3.1 days**	1.7 days

*:p < 0.05; **:p < 0.01.

In all three virus subgroups, a significant reduction in duration of common cold was recorded in carrageenan treated patients compared to placebo ones in ITT population. Similarly, a significant reduction was seen in PP population between carrageenan and placebo groups except for InfA patients.

### Relapses in subgroups

**Figure 6:**
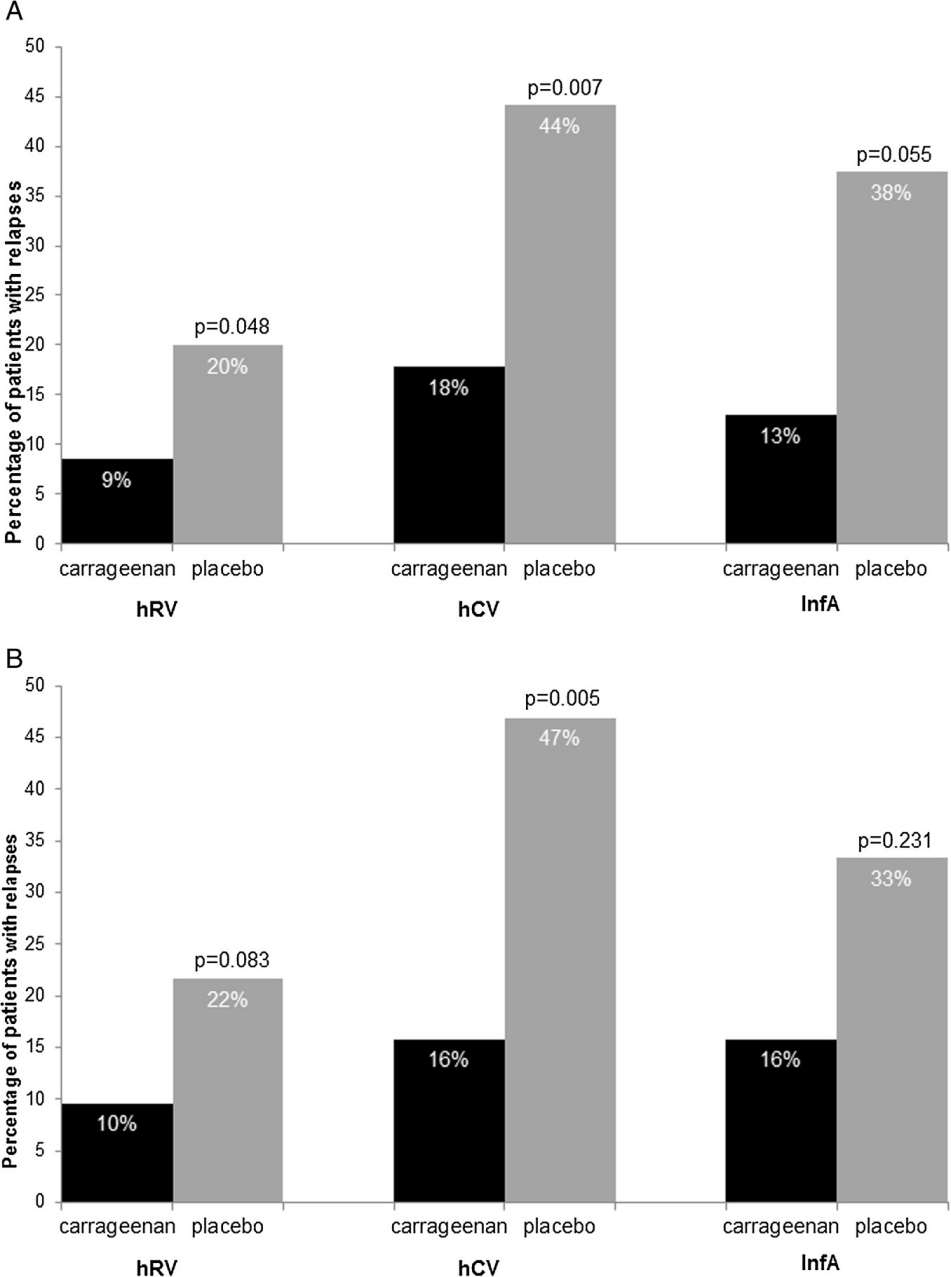
**Percentage of patients suffering from a relapse during 21 days of observation period. A**: ITT population (hRV: carrageenan n = 70, placebo n = 80; hCV: carrageenan n = 45, placebo n = 43; InfA: carrageenan n = 45, placebo n = 43) **B**: PP population (hRV: carrageenan n = 52, placebo n = 60; hCV: carrageenan n = 38, placebo n = 32; InfA: carrageenan n = 19, placebo n = 15).

Relapses occurred significantly more frequently in placebo groups of hRV and hCV infected patients compared to carrageenan group, in ITT population. Similarly, in InfA infected patients a trend to more relapses was observed in placebo group (p = 0.055) (Figure 6A). Also in the PP population, a higher percentage of relapses was noticed in placebo treated virus infected subgroups. However, only in hCV infected patients this difference reached statistical significance (p = 0.005) (Figure 6B). In the ITT population, the average number of relapses per patient was significantly lower in carrageenan treated virus-infected subgroups. Thus, in hRV infected patients, the average number of relapses per patient was 0.13 in carrageenan treated patients and 0.79 in placebo treated ones (p = 0.04). In hCV infected patients relapses per patient occurred 0.2 times in carrageenan treated patients compared with 0.58 times in placebo treated ones (p = 0.007). Moreover, placebo treated InfA infected patients had 0.79 relapses per patient compared to 0.13 relapses per patient in carrageenan group (p = 0.04).

Likewise, to total relapses in PP population, a higher average number of relapses per patient was recognized in all virus infected subgroups but solely in hCV infected subpopulation this difference reached significance (0.18 versus 0.53, p = 0.006).

### Safety

Overall tolerability of study products was very good and there was no difference between treatment arms in the number of patients with AEs or severe adverse events (SAEs) (Table 7). About 80% of patients did not report any AE until the end of the observation period. Interestingly, rhinitis (newly diagnosed cases and deterioration of the condition during the study) was observed more frequently in the placebo group (no case in carrageenan treatment arm compared to 4 cases in placebo), with a statistically significant difference between groups (p = 0.045). In the two studies six patients experienced SAEs, none of them was study related and all SAEs resolved until the end of the study. All AEs were rated as not-study-related.

**Table 7:** **Overview of most commonly reported AEs**

	Carrageenan (n = 126)		Placebo (n = 128)		Total (n = 254)	
	n	%	n	%	n	%
N. of patients without AEs	103	81,7%	99	77,3%	202	79,5%
Cough	1	0,8%	4	3,1%	5	2,0%
Rhinitis*	0	0,0%	4	3,1%	4	1,6%
Otitis media	3	2,4%	1	0,8%	4	1,6%
Nasal bleeding	3	2,4%	1	0,8%	4	1,6%
Fever, infection NOS**	2	1,6%	2	1,6%	4	1,6%
Bronchitis	2	1,6%	2	1,6%	4	1,6%
Nasal disorders NOS***	1	0,8%	2	1,6%	3	1,2%
Vomiting	1	0,8%	1	0,8%	2	0,8%
Tonsillitis	2	1,6%	0	0,0%	2	0,8%
Sinusitis	0	0,0%	2	1,6%	2	0,8%
Rash	0	0,0%	2	1,6%	2	0,8%
Nausea	0	0,0%	2	1,6%	2	0,8%
Ear pain	1	0,8%	1	0,8%	2	0,8%
Conjunctivitis	1	0,8%	1	0,8%	2	0,8%
Adenoids	1	0,8%	1	0,8%	2	0,8%
Abdominal pain	1	0,8%	1	0,8%	2	0,8%

*Difference between groups statistically significant, p = 0.045.

**Including pyrexia, febrile infection and viral infection (not specified).

***Including nasal itching, feeling of burning in the nose and smell disturbance.

NOS = not specified.

## Discussion

In the present study data from two randomized controlled clinical trials, investigating the effectiveness of carrageenan nasal spray during virus-confirmed common cold, were analyzed. This analysis showed that carrageenan-containing nasal spray reduced the duration of viral-confirmed common cold by almost 2 days. Furthermore, carrageenan-treated patients cleared more than twice as many viruses when compared to placebo-treated patients. Additionally, more virus-free patients were found in carrageenan-treated patients at visit 2. Another clinical benefit of carrageenan nasal spray was the significant reduction in relapses defined as recurrence of symptoms after a patient was free of symptoms for at least one day.

Annually common colds account for more than 20 million doctor visits and 40 million lost school and work days in the USA alone [5]. It is the most frequently observed infectious disease in human beings, with four to eight episodes per year in children and three to five in adults [1, 17]. Therefore, a reduction of disease duration by 2 days has an enormous impact on economic losses and social welfare of children and their families.

Common cold is mainly caused by respiratory viruses, such as human rhinovirus, human coronavirus, parainfluenza, influenza, respiratory syncytial virus, adenovirus, enterovirus and metapneumovirus [4, 5, 18, 19]. Since it is impossible for an individual to determine the cause of a cold, it is important to know if the product used provides effectiveness against many different respiratory viruses causing colds. In the present study, the analysis of virus species revealed that human rhinovirus, human coronavirus and influenza A virus were the most common species. Nasal administration of carrageenan during common cold episodes consistently showed a reduction in duration of disease in patient subgroups infected with these three viruses. The decrease in duration of disease varied between 1.9 days for rhinovirus up to 3.9 days for coronavirus with infA lying in between with a reduction of 3.3 days (Table 6). The study data suggest that carrageenan nasal spray exerts an unspecific effect on viruses of different etiologic origin, both enveloped and non-enveloped viruses. Experimental data support this notion showing that carrageenan exhibited a potent inhibitory effect on papilloma virus [9]. Moreover, *in vitro* and *in vivo* studies revealed that carrageenan was effective against several human respiratory viruses such as influenza A [11] human rhinovirus [10], respiratory syncytial virus and also of human enterovirus 71 [12]. It was shown that the carrageenan polymer directly bound to the virus and prevented attachment of virus to the cells [11]. Hence, carrageenan works via an unspecific physical mode of action at a very early stage of virus life cycle.

As common cold is a benign self-limiting disease, the decrease in viral titers between visit 1 and visit 2 in carrageenan-treated and in placebo-treated patients was expected. But carrageenan-treated patients cleared more than twice as many viruses compared to placebo-treated patients leading to more virus-free patients at visit 2 in carrageenan group (Table 4). It can be concluded that treatment with iota-carrageenan results in a significant reduction of the viral load and the number of detectable viruses already at days 3–7 (V2). Absence of viruses leads to a faster recovery and hence earlier clearance of symptoms. These data are in line with the results of all currently available clinical trials regarding iota-carrageenan-treatment during common cold [13–15]. A further benefit of carrageenan treatment was the significant reduction in relapses defined as recurrence of symptoms after a patient was free of symptoms for at least one day (Figure 3). Because most relapses took place after cessation of treatment a prophylactic effect of carrageenan nasal spray could be hypothesized. Similarly to all virus positive patients, relapses were reduced by carrageenan nasal spray in all three virus-infected patient subgroups. Again, patients infected with human coronavirus benefited most from carrageenan treatment showing a 2.5-fold reduction in relapses compared to placebo (Figure 6). Human rhinovirus infected patients as well as influenza A infected patients revealed a twofold reduction in relapses when treated with carrageenan nasal spray.

The reduction of relapses and the reduction of virus titer in carrageenan treated patients might be of interest in particular patient subgroups with underlying diseases such as asthma and COPD. Numerous recent reviews discuss the presence of respiratory viruses as cause of exacerbations of asthma and COPD [20–22]. Acute exacerbations are associated with decreased lung growth in children or accelerated loss of lung function and, as such, add substantially to both the cost and morbidity associated with asthma [23]. Therefore, a reduction of virus load and persistence should have a beneficial effect for these patients. Especially children who frequently suffer from viral infections often involving more than one virus could benefit from the carrageenan nasal spray. Due to the physical mode of action and the favorable side effect profile, it could be used as a cost-effective long time preventive measure. The cost for treatment lies below one dollar per day. Further clinical studies should be done to investigate the prophylactic effect of the carrageenan nasal spray in patients with respiratory diseases as well as other patients groups where the prevention of viral infections is of utmost importance (e.g. cancer patients, immunocompromised patients).

The limitations of this study include the retrospective nature of the data analysis, while some additional clinical information of the patients would have been necessary for underlining our findings. Furthermore, the clinical benefit of using carrageenan nasally was not measured e.g. by spirometry. However, in small children included in the data set, it is not always possible to perform spirometric measurements.

## Conclusions

In conclusion, nasal application of carrageenan spray in children as well as in adults suffering from virus-confirmed common cold reduced duration of disease, increased viral clearance and reduced relapses. Therefore, carrageenan nasal spray can be regarded as a safe and effective treatment with a high potential for reducing social and economic burden caused by common cold.
